# Supplementary material for: The interactome of intact mitochondria by cross-linking mass spectrometry provides evidence for coexisting respiratory supercomplexes
Source: Mol Cell Proteomics. 2017 Dec 8;17(2):216–32. doi: 10.1074/mcp.RA117.000470 (PMC5795388; doi:10.1074/mcp.RA117.000470)
Supplement: Supplemental Data [file supp_RA117.000470_133922_0_supp_23978_fzffwf.zip › spectra_annotation/mito_DR_spectra_annotation/83-1-8-1-9-1.pdf]

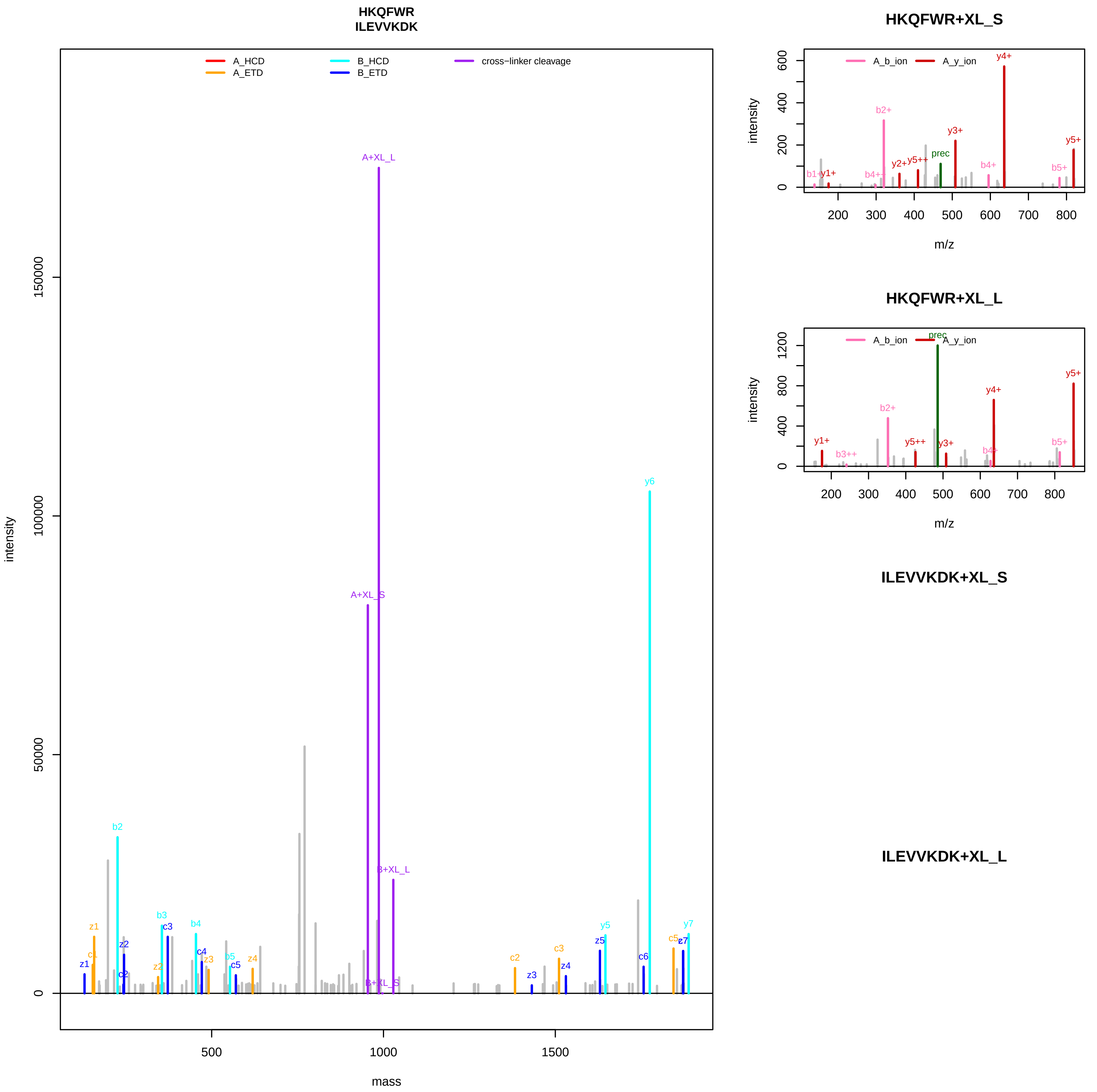

HKQFWR
ILEVVKDK
A_HCD
A_ETD
B_HCD
B_ETD
cross−linker cleavage
A+XL_L
A+XL_S
B+XL_L
B+XL_S
y6
y7
y5
z5
c6
z7
c5
c3
z4
c2
z3
b2
b3
c3
b4
c4
b5
c5
z1
c1
z2
c2
z3
z4
intensity
mass
0
50000
100000
150000
500
1000
1500
HKQFWR+XL_S
A_b_ion
A_y_ion
b1+
y1+
b2+
b4++
y2+
y5++
y3+
prec
y4+
b4+
b5+
y5+
intensity
m/z
0
200
400
600
200
300
400
500
600
700
800
HKQFWR+XL_L
A_b_ion
A_y_ion
prec
y1+
b3++
b2+
y5++
y3+
y4+
b4+
b5+
y5+
intensity
m/z
0
400
800
1200
200
300
400
500
600
700
800
ILEVVKDK+XL_S
ILEVVKDK+XL_L
